# Is postmenopausal endometrial fluid collection alone a risk factor for endometrial cancer?

**DOI:** 10.12669/pjms.341.13990

**Published:** 2018

**Authors:** Gulin Feykan Yegin Akcay, Emre Erdem Tas, Ayse Filiz Yavuz

**Affiliations:** 1 Gulin Feykan Yegin Akcay, MD. Department of Gynecology and Obstetrics, Ankara Ataturk Education and Research Hospital, Ankara, Turkey; 2 Emre Erdem Tas, Assistant Professor, Department of Gynecology and Obstetrics, Yildirim Beyazit University, Ankara, Turkey; 3 Prof. Ayse Filiz Yavuz, Department of Gynecology and Obstetrics, Yildirim Beyazit University, Ankara, Turkey

**Keywords:** Menopause, Ultrasonography, Endometrium, Uterine neoplasms

## Abstract

**Objective:**

To determine the usefulness of single-layer, ultrasonographic measurement of endometrial fluid collection (EFC) volume to predict endometrial pathology in asymptomatic postmenopausal patients.

**Methods:**

One hundred fifty asymptomatic postmenopausal women were analysed retrospectively from January 2012 to December 2016. After patients with endometrial hyperplasia/neoplasia were included in Group-I, and those with insufficient tissue, endometrial atrophy, or endometritis were included in Group-II; Groups one and two were compared with respect to primary (correlations between endometrial thickness and EFC volume) and secondary (correlations between demographic characteristics and EFC volume) outcomes.

**Results:**

There was no correlation between EFC volume and single-layer endometrial thickness (*P* = 0.36). Likewise, demographic characteristics were not related to EFC (*P* > 0.05). However, both EFC volume and single-layer endometrial thickness were thicker in Group-I compared to Group-II (4.8 ± 1.9 mm *vs*. 3.7 ± 2.5 mm; and 5.7 ± 9.4 mm *vs*. 2.7 ± 2.5 mm, respectively) (*P* values were < 0.05).

**Conclusion:**

Although a cutoff value for endometrial thickness and EFC volume could not be recommended based on our study findings, it should be noted that 2% is a clinically significant rate of malignancy. Thus, postmenopausal patients with EFC should be evaluated for endometrial sampling.

## INTRODUCTION

Imaging advances have allowed ultrasound to play an increasingly important role in early diagnosis of uterine and adnexal pathology. However, the finding of endometrial fluid collection (EFC) in asymptomathic, postmenopausal women is still a confusing sign for gynecologists. EFC is often an incidental finding on ultrasounds, with an incidence of 4% to 14%.^1^ It has been suggested that a single layer endometrial thickness of 4 mm should be the cutoff value beyond which above endometrial sampling should be performed, regardless of EFC presence, and that the surrounding endometrium and its thickness should be a more important consideration than presence of fluid alone.^2^,^3^ However, several studies have shown that the presence of EFC may indicate endometrial and cervical pathology.^4^,^5^ Indeed, some investigators have reported cases of endometrial carcinoma that presented ultrasonographically with a thin endometrium in the presence of EFC.^6^-^8^

Management of asymptomatic, postmenopausal women with EFC is unclear, and the utility of routine endometrial sampling has been debated in these cases because of the expense and invasiveness of these procedures. The aim of this study was to determine the usefulness of single-layer, ultrasonographic measurement of EFC volume to predict endometrial pathology in asymptomatic postmenopausal patients.

## METHODS

Of the 2455 asymptomatic postmenopausal women receiving care at our clinic between January 2012 and December 2016, there were 174 women who had EFC during transvaginal ultrasonography and underwent endometrial sampling. Of those women, 150 patients with no history of cervical excisional procedure, tamoxifen use, hormone replacement therapy use, or abnormal cytology on PAP test were included in the study. Patient data were obtained retrospectively from medical records at our hospital. This study was approved by the Ethical Review Board Committee (Approval No:) of Ankara Yildirim Beyazit University Faculty of Medicine (Ankara, Turkey). Menopause was defined as at least 12 months of amenorrhea in a woman over the age of 40. The thickest endometrial portion, as viewed in the sagittal uterine plane, was recorded as the single-layer endometrial thickness. The thickest anechoic area, also viewed in the sagittal uterine plane, was recorded as the EFC volume. A 2D grey-scale Logiq 200 PRO Ultrasound Device (GE Medical Systems, Milwaukee, USA) with a 7.5 mHz endovaginal probe was used by two experienced clinicians.

Primary outcome measures were correlations between endometrial thickness and EFC volume in asymptomatic postmenopausal women. Secondary outcome measures were related to possible correlations between demographic characteristics and EFC volume. Age, gravidity, parity, menopausal duration, EFC volume, endometrial thickness, and endometrial sampling were evaluated. Endometrial sampling was performed using a Pipelle device, and dilatation and curettage was required for two patients due to cervical stenosis. After patients with endometrial hyperplasia or neoplasia were included in Group-I, and those with insufficient tissue, endometrial atrophy, or endometritis were included in Group-II; Groups 1 and 2 were compared with respect to primary and secondary outcomes.

Statistical analyses were performed using SPSS version 21.0 (IBM Corp., Armonk, NY, USA). The Kolmogorov–Smirnov test was used to assess the normality of data. Normally distributed data were expressed as means ± standard deviations and ranges, whereas non-parametric data were expressed as medians, interquartile ranges, and ranges. The independent samples *t* test and Mann–Whitney *U* test were used to compare parametric and non-parametric data, respectively, between groups. Degrees of association between EFC volume and single-layer endometrial thicknesses were calculated using Spearman's rank correlation coefficient. Receiver operating characteristic (ROC) curves were used to determine the relationship between both EFC volume and single-layer endometrial thickness for both groups. A *P* value of < 0.05 was considered statistically significant for all tests.

## RESULTS

Clinical, ultrasonographic, and histopathologic characteristics for all patients with EFC, based on group are summarized in Table-I. Final endometrial histopathologic examination showed malignant neoplasms in three patients with EFC (2%). One patient who had endometrial intraepithelial neoplasia underwent hysterectomy; intraoperative frozen-section analysis and final pathologic examination confirmed a diagnosis of endometroid type endometrial cancer. The two other patients were diagnosed with endometroid type endometrial cancer and uterine carcinoma, respectively.

**Table-I:** Demographic, clinical and histopathologic characteristics of all women with endometrial fluid, overall and according to groups (Group-I and 2).

Characteristics	All patients (*n*=150, 100%)	Group-I (*n*=8, 5.3%)	Group-II (*n*=142, 94.7%)	P
*Demographic*				
Age (years), mean ± SD (range)	59.9±8.7 (43-83)	63±9.3 (50-77)	59±8.7 (43-83)	0.28
Gravidity, median (IQR) (range)	4 (2) (0-12)	3.5 (6.75) (0-9)	4 (2) (0-12)	0.58
Parity, median (IQR) (range)	3 (2) (0-10)	2.5 (5.25) (0-8)	3 (2) (0-10)	0.34
Duration of menopause (year), median (IQR) (range)	9 (13) (1-40)	16 (19.5) (1-27)	9 (13) (1-40)	0.67
*Clinical*				
Endometrial fluid (mm), mean ± SD (range)^α^	2.9±3.3 (1-29)	5.7±9.4 (1.5-29)	2.7±2.5 (1-20)	0.017
Single-layer endometrial thickness (mm), mean ± SD (range)^α^	3.7±1.4 (2-8)	4.8±1.9 (2 - 8)	3.7±1.3 (2 - 8)	0.01
*Histopathological (n, %)*				
Insufficient tissue	76 (50.7 %)			
Endometrial atrophy	63 (42 %)			
Endometrial polyp	5 (3.3 %)			
Endometritis	3 (2%)			
Endometroid Type Endometrial Cancer	2 (1.3%)			
Uterine Carcinosarcoma	1 (0.7%)			

*Footnote:* Patients with endometrial hyperplasia or neoplasia were included in Group-I, and those with insufficient tissue, endometrial atrophy, or endometritis were included in Group-II.

α Endometrial fluid volume and single-layer endometrial thickness were measured as the thickest form viewed in the sagittal uterine plane.

*Abbreviations:* SD, standard deviation; IQR, interquartile range.

**Fig. 1:**
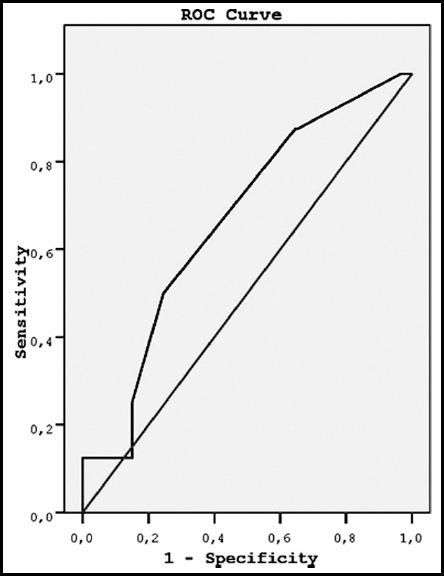
Receiver operating characteristic (ROC) curve analysis of the relationship between endometrial fluid and group. AUC = area under the curve = 0.65; standard error = 0.092; P=.09; 95% confidence interval = 0.48 to 0.84

**Fig. 2:**
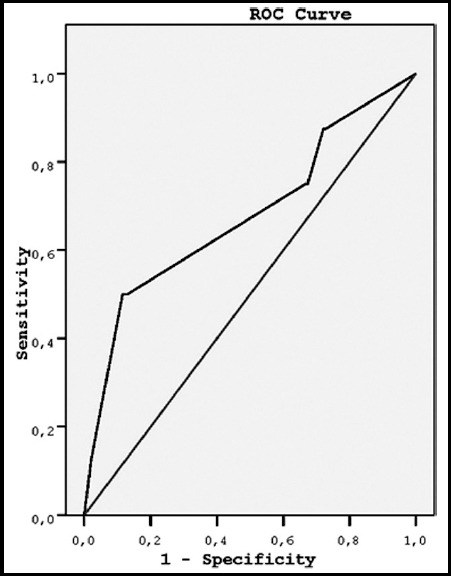
Receiver operating characteristic (ROC) curve analysis of the relationship between single-layer endometrial thickness and group. AUC = area under the curve = 0.68; standard error = 0.1; P=.1; 95% confidence interval = 0.45 to 0.9

There was no correlation between EFC and single-layer endometrial thickness (Spearman's correlation coefficient, *P* = 0.36). Likewise, demographic characteristics were not related to EFC (*P* values were > 0.05). However, both EFC volume and single-layer endometrial thickness were larger in Group-I compared to Group-II (4.8 ± 1.9 mm vs 3.7 ± 2.5 mm; and 5.7 ± 9.4 mm vs 2.7 ± 2.5 mm, respectively) (*P* values were < 0.05) (Table-I). However, ROC curve analyses did not differ between groups with respect to EFC volume (*P* = 0.09) or single-layer endometrial thickness (*P* = 0.1) (Fig.1 and 2).

## DISCUSSION

The reported incidence of EFC on sonography in postmenopausal women varies from 4% to 14%.^1^ However, studies of EFC in asymptomatic, postmenopausal women are limited and conflicting. Some investigators have reported endometrial cancer with EFC as an incidental finding in the absence of endometrial thickening.^6^-^8^ In contrast, Krissi et al have recommended endometrial sampling for all patients with EFC, regardless of endometrial thickness. Their hypothesis was that fluid can mask true pathology by exerting pressure on the endometrial lining.^7^ Another study asserts that criteria such as endometrial lining smoothness and symmetry should be used to influence decisions to histologically examine all cases irrespective of endometrial thickness.^9^

Researchers who have studied patient outcomes in the context of EFC with endometrial thickening have recommended 3 mm or 4 mm as the cutoff value requiring further investigation.^1^,^2^,^9^ Obtaining a 3-month reference sonogram follow-up has also been recommended for women with inconspicuous thin endometria.^8^ However, some authors assert that EFC may be associated with malignancy even in the presence of endometria measuring less than 4mm.^3^-^5^ Another study group recommended endocervical sampling in asymptomatic, postmenopausal patients with EFC, even when endometrial thickness is 3mm or less; endometrial sampling was also recommended for patients with echogenic EFC and any amount of endometrial thickening.^10^ The most striking finding about the relationship between EFC and malignancy was reported by Breckenridge et al, who showed that uterine or cervical carcinoma occurred in 16 of 17 patients (94%) with EFC.^4^

In our study, endometrial thickness and EFC were higher in patients with hyperplastic and neoplastic pathology, but this relationship cannot be shown using ROC curve analyses. Of 150 patients, three (2%) had malignancies of the uterine corpus or endometrium. However, this correlation with endometrial cancer and EFC was not as high as shown previously, nor as low as shown recently.^1^,^4^

### Limitations of the study

Our study is limited by its retrospective and single-centered design; thus, prospective randomized studies with larger sample sizes are required to verify our results.

## CONCLUSION

Although a cutoff value for endometrial thickness and EFC could not be recommended based on our study findings, it should be noted that 2% is a clinically significant rate of malignancy. Thus, postmenopausal patients with EFC should be evaluated for endometrial sampling. Further studies are needed to evaluate cost effectiveness before routine endometrial sampling can be recommended for asymptomatic postmenopausal women with endometrial fluid collection.

### Authors' Contributions

**GFYA** conceived, designed and did statistical analysis & editing of manuscript.

**GFYA, EET and AFYA** did data collection and manuscript writing.

**GFYA** takes the responsibility and is accountable for all aspects of the work in ensuring that questions related to the accuracy or integrity of any part of the work are appropriately investigated and resolved.
